# Ryanodine receptors contribute to the induction of nociceptive input-evoked long-term potentiation in the rat spinal cord slice

**DOI:** 10.1186/1744-8069-6-1

**Published:** 2010-01-20

**Authors:** Long-Zhen Cheng, Ning Lü, Yu-Qiu Zhang, Zhi-Qi Zhao

**Affiliations:** 1 Institute of Neurobiology, Institutes of Brain Science and State Key laboratory of Medical Neurobiology, Fudan University, Shanghai 200032, China

## Abstract

**Background:**

Our previous study demonstrated that nitric oxide (NO) contributes to long-term potentiation (LTP) of C-fiber-evoked field potentials by tetanic stimulation of the sciatic nerve in the spinal cord *in vivo*. Ryanodine receptor (RyR) is a downstream target for NO. The present study further explored the role of RyR in synaptic plasticity of the spinal pain pathway.

**Results:**

By means of field potential recordings in the adult male rat *in vivo*, we showed that RyR antagonist reduced LTP of C-fiber-evoked responses in the spinal dorsal horn by tetanic stimulation of the sciatic nerve. Using spinal cord slice preparations and field potential recordings from superficial dorsal horn, high frequency stimulation of Lissauer's tract (LT) stably induced LTP of field excitatory postsynaptic potentials (fEPSPs). Perfusion of RyR antagonists blocked the induction of LT stimulation-evoked spinal LTP, while Ins(1,4,5)P3 receptor (IP_3_R) antagonist had no significant effect on LTP induction. Moreover, activation of RyRs by caffeine without high frequency stimulation induced a long-term potentiation in the presence of bicuculline methiodide and strychnine. Further, in patch-clamp recordings from superficial dorsal horn neurons, activation of RyRs resulted in a large increase in the frequency of miniature EPSCs (mEPSCs). Immunohistochemical study showed that RyRs were expressed in the dorsal root ganglion (DRG) neurons. Likewise, calcium imaging in small DRG neurons illustrated that activation of RyRs elevated [Ca^2+^]_i _in small DRG neurons.

**Conclusions:**

These data indicate that activation of presynaptic RyRs play a crucial role in the induction of LTP in the spinal pain pathway, probably through enhancement of transmitter release.

## Background

LTP is a long-lasting form of synaptic plasticity in many parts of the central nervous system, particularly in the hippocampus [1]. Likewise, compelling evidence reveals that tetanic stimulation of the peripheral nerve produces LTP of C-fiber-evoked responses in the spinal dorsal horn both *in vivo *[2-5] and *in vitro *[6,7], and prolonged behavioral pain hypersensitivity [8]. Therefore, it is conceivable that C-afferent-induced spinal LTP may be a substrate for central sensitization of the pain pathway, which amplifies nociceptive input resulting in hyperalgesia [4].

Nitric oxide (NO) is a diffusible messenger throughout the central nervous system. Considerable evidence implicates the formation of NO in mechanisms underlying hyperalgesia and allodynia [9-11]. Likewise, NO contributes to spinal LTP of C-fiber-evoked field potentials [12-14]. This gas may diffuse retrogradely from postsynaptic neurons of the spinal dorsal horn, enhancing the release of pain-associated transmitters. It is, therefore, speculated that the presynaptic action of NO may contribute to the induction of spinal LTP [12,13]. To our knowledge, there is no direct morphological and physiological evidence to identify the action of NO on nociceptive primary afferent terminals in the spinal cord.

It is known that the ryanodine receptor (RyR) is a down-stream target for NO [15]. The RyR and the Ins(1,4,5)P3 receptor (IP_3_R) in intracellular calcium stores constitute a fundamental calcium source. Ca^2+ ^released from calcium stores via RyRs is implicated in transmitter release from presynaptic terminals of central synapses [16,17].

Using spinal cord slice preparations and field potential and patch-clamp recordings from superficial dorsal horn neurons, field potential recordings in the spinal dorsal horn *in vivo*, immunohistochemistry and calcium imaging in DRG neurons, the present study was to address a possible role of ryanodine receptors in the induction of spinal LTP.

## Results

### LTP of fEPSPs by stimulation of Lissauer's tract (LT) in the spinal cord slice

**Figure 1:**
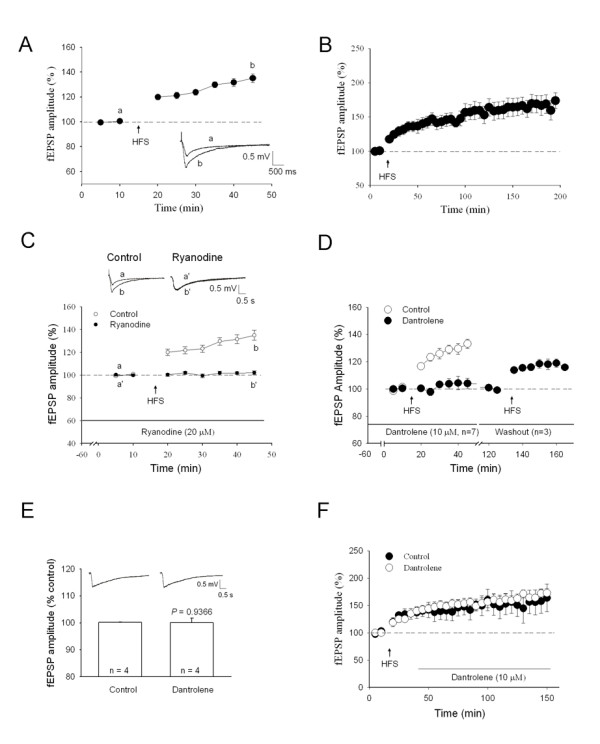
**Involvement of RyR in the induction of LTP of fEPSPs in the spinal dorsal horn *in vitro***. ***A***, fEPSPs were recorded in the presence of 10 μM bicuculline methiodide and 1 μM strychnine. fEPSPs recorded 10 min before HFS served as control. After HFS, fEPSPs significantly increased (n = 24). The inserted traces were the original recordings (without averaging). a-b corresponds to the time points as indicated. ***B***, The LTP of fEPSPs stably lasted for 3 h (n = 8). ***C***, 20 μM ryanodine applied 1 h before HFS blocked the induction of LTP (*P *< 0.001) (control n = 7; ryanodine n = 8). The inserted traces were the original recordings. ***D***, 10 μM dantrolene perfused 1 h before HFS blocked the induction of LTP (*P *< 0.001) (control n = 7; dantrolene n = 7), and this effect was reversible (*P *< 0.001) (washout n = 3). ***E***, 10 mM dantrolene has no significant effect on the amplitude of fEPSP. Control: the amplitude of fEPSP recorded 5 min before dantrolene application (10 μM); Dantrolene: 30 min after perfusion of dantrolene. Inserts show original traces recorded 5 min before and 30 min after dantrolene. ***F***, 10 μM dantrolene perfused 30 min after HFS had no significant effect on the established LTP (*P *= 1) (control n = 5; dantrolene n = 6). Data were shown as mean ± s.e.m. in all figures; *n*: the number of slices.

Field potentials in the spinal dorsal horn (DH) were induced by stimulation of the LT (0.1 ms, 0.7-1 mA, 5-min interval) via a bipolar tungsten electrode. After perfusion with bicuculline methiodide (10 μM) and strychnine (1 μM) for 1 h, field excitatory postsynaptic potentials (fEPSPs) recorded stably for 10 min served as control. Following high-frequency stimulation (HFS, 100 Hz for 1 sec, three times at 10-sec intervals), fEPSPs were significantly enhanced to 132.4 ± 3.1% of control (*P *< 0.001, Fig. 1A) at 30 min after HFS in all 24 recorded cases, indicating creation of spinal LTP. The LTP of fEPSPs stably lasted for more than 3 h (173.9 ± 11.4% of control, n = 8, *P *< 0.001) (Fig. 1B).

### Involvement of RyR, but not IP3R, in the induction of spinal LTP

It is reported that low (2 μM) and high (20 μM) concentrations of ryanodine function as agonist and antagonist of RyRs in the hippocampus, respectively [18]. Perfusion with 20 μM ryanodine 1 h before conditioning HFS failed to enhance fEPSPs (102.1 ± 1.6% of control, n = 8; Fig. 1C). Similarly, dantrolene (10 μM), another antagonist for RyR, also did not evoke significant potentiation of fEPSPs (104.0 ± 3.8% of control, n = 7; Fig. 1D) without the effect on the baseline of fEPSPs (Fig. 1E). With washout of dantrolene, spinal LTP returned (119.0 ± 3.1% of control, n = 3; Fig. 1D). These results indicated blockade of the induction of LTP by RyR antagonists. It is reasonable to conclude that RyRs are involved in the induction of LTP of fEPSPs. However, When dantrolene was sustainedly delivered 30 min after conditioning HFS, the established LTP was not blocked (164.1 ± 5.6% of control, n = 6; Fig. 1F), suggesting that RyRs are unlikely to contribute to the maintenance of spinal LTP.

**Figure 2:**
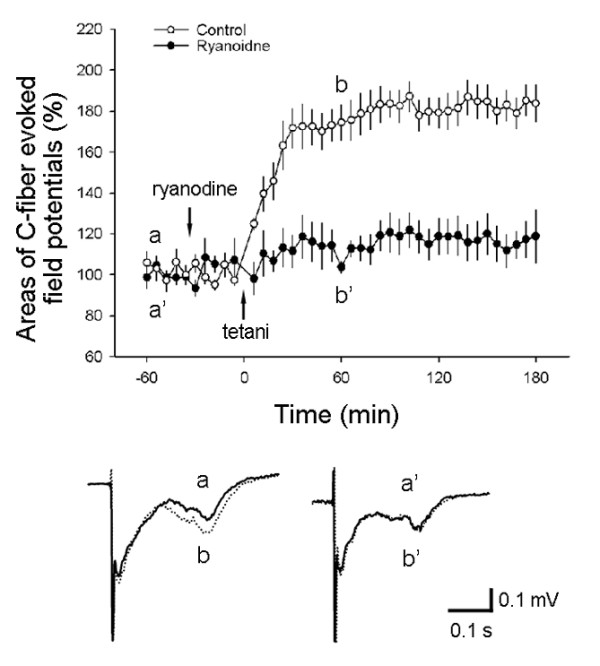
**Involvement of RyR in the induction of LTP of fEPSPs in the spinal dorsal horn *in vivo***. The spinal LTP was blocked by high concentration of ryanodine (1 mM, 10 μl) intrathecally applied 30 min before tetanic stimulation (*P *< 0.001, *vs*. vehicle) (control n = 5; ryanodine n = 6) in the spinal cord *in vivo*. Representative original recordings (average of six consecutive traces) were shown below from the time points as indicated.

To further confirm the involvement of RyR in the induction of LTP in the spinal pain pathway, we then performed electrophysiological recordings *in vivo*. Consistent with our previous study [3], C-fiber-evoked field potentials in the spinal dorsal horn were induced by electrical test stimulation of the sciatic nerve (0.5 ms, 15-20 V, 1-min intervals) in anesthetized rats. When tetanic stimulation (0.5 ms, 100 Hz, 30-40 V, 10 trains of 2-sec duration at 10-sec intervals) was applied to the sciatic nerve 30 min after intrathecal injection of vehicle (0.25% DMSO, 10 μl), LTP of C-fiber-evoked field potentials was induced (*P *< 0.001, compared with the baseline responses of 30 min before intrathecal injection of vehicle). The field potentials were increased to 174.46 ± 8.66% of control at 1 h after tetanic stimulation. When ryanodine (1 mM, 10 μl) was intrathecally applied 30 min before tetanic stimulation, the induction of spinal LTP was prevented (*P *< 0.001, compared with vehicle, Fig. 2).

**Figure 3:**
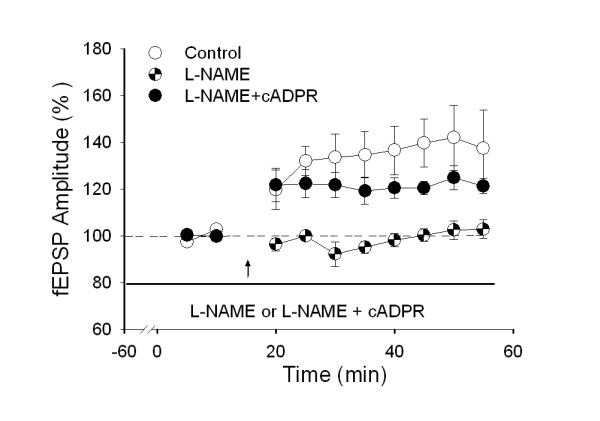
**Reversal of L-NAMA-induced inhibition of LTP of fEPSP by cADPR, an endogenous agonist of the RyR**. Perfusion with 50 μM L-NAME for 1 h before HFS of LT inhibited the induction of LTP (*P *< 0.001), which was reversed by co-application of L-NAME and 5 μM cADPR for 1 h before HFS (*P *< 0.001) (control, n = 5; L-NAME, n = 6; L-NAME + cADPR, n = 5).

The RyR is a downstream target of NO [15]. Perfusion with 50 μM L-NAME, a NO synthase (NOS) inhibitor, 1 h before conditioning HFS of LT inhibited the induction of LTP (Fig. 3). If the RyR as a NO target was activated, L-NAME-induced inhibition of spinal LTP might be reversed and in turn LTP could occur. As expected, when 5 μM cyclic ADP-ribose (cADPR), an endogenous agonist of the RyR, was perfused 10 min before L-NAME, LTP re-appeared (Fig. 3).

**Figure 4:**
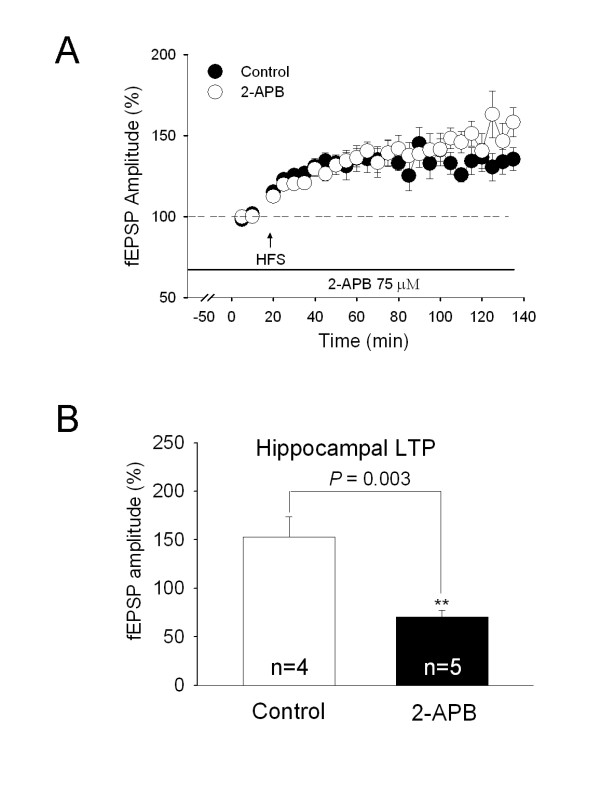
**No effect of IP3R antagonist on induction of spinal LTP *in vitro***. ***A***, IP_3_R antagonist 2-APB (75 μM) applied for 1 h before HFS of LT had no significant effect either on the induction of LTP of fEPSPs (*P *= 0.303) (control, n = 7; 2-APB, 45 min from baseline, n = 8), or on the established LTP of fEPSPs (*P *= 0.173) (control, n = 4; 2-APB, 135 min from baseline, n = 4). ***B***, As a positive control, 2-APB blocked the induction of hippocampal LTP. In the hippocampal slice, 75 μM 2-APB perfused for 1 h before conditioning stimulation (100 Hz for 1 sec, 3 times at 10-sec intervals, mimicking spinal HFS) blocked the induction of hippocampal LTP (control, 30 min after induction of hippocampal LTP in the presence of 10 μM bicuculline methiodide, n = 4; 2-APB, 30 min after induction of hippocampal LTP in the presence of 10 μM bicuculline methiodide and 75 μM 2-APB, n = 5). Error bars represent s.e.m. n: the number of slices. **P < 0.01.

We detected possible effect of another calcium store receptor IP_3_R on spinal LTP. When 75 μM 2-APB, an IP_3 _receptor antagonist, was perfused 1 h before HFS, there was no significant effect on the induction of LTP (126.4 ± 4.2% of control, n = 8; Fig. 4A). Moreover, sustained perfusion with 2-APB (75 μM) 1 h before conditioning HFS of LT and for the whole period of recording failed to alter the established LTP (Fig. 4A) (158.3 ± 8.9% of control, n = 4). To exclude whether the negative result may be attributable to the low efficacy of 2-APB, we further assessed its effect in hippocampal slices. The amplitude of fEPSPs were increased to 154.1 ± 21.2% of control at 30 min after conditioning HFS (n = 4). Perfusion with 75 μM 2-APB 1 h prior to HFS completely blocked the induction of hippocampal LTP (67.4 ± 10.2% of control, 30 min after HFS; n = 5) (Fig. 4B). This result provides additional evidence for the lack of involvement of IP_3 _receptors in both the induction and maintenance of spinal LTP.

### RyRs agonist alone is sufficient for inducing LTP of fEPSPs in spinal cord slice

**Figure 5:**
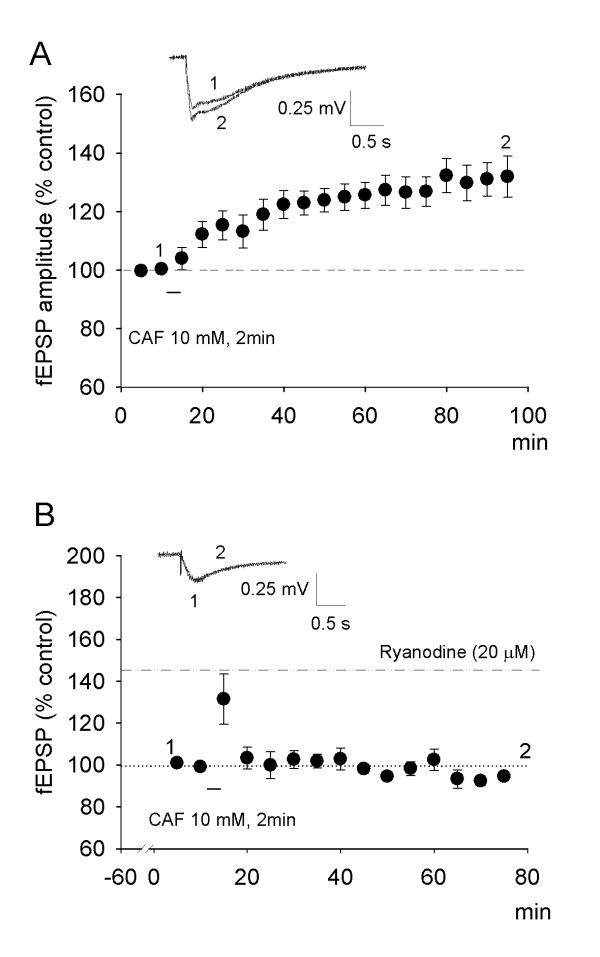
**Caffeine-induced LTP of fEPSPs in spinal cord slice**. ***A***, After perfusion with 10 mM caffeine for 2 min instantly following washed out, LTP of fEPSPs occurred without conditioning stimulation in the presence of 10 μM bicuculline methiodide and 1 μM strychnine, which lasted for more than 80 min. Inserts show original recordings 5 min before (1) and about 80 min after (2) 10 mM caffeine challenge. Traces were not averaged (n = 13). ***B***, Perfusion with 20 μM ryanodine 1 hour before caffeine challenge blocked the induction of LTP of fEPSPs. Inserts show original recordings without averaging (n = 5).

Field potentials were evoked by stimulation of the dorsal root (DR) in the spinal cord slice with an attached dorsal root. Caffeine at 10 mM, RyR agonist, can activate Ca^2+^-induced Ca^2+ ^release by increasing the affinity for Ca^2+ ^of the RyRs [19]. To address whether activation of RyRs is sufficient for induction of spinal LTP, we tested the effect of 10 mM caffeine on DR stimulation-induced fEPSPs in the presence of 10 μM bicuculline and 1 μM strychnine. Perfusion with 10 mM caffeine alone for 2 min without conditioning HFS produced LTP of fEPSPs lasting for more than 80 min, during which the amplitude of fEPSPs increased to 131.9 ± 7.0% of control (n = 13, Fig. 5A). Notably, the potentiation was greater after caffeine washout for about 30 min. When 20 μM ryanodine was perfused for 1 h before caffeine challenge to inactivate RyRs, the enhancement of fEPSPs was completely blocked (92.4 ± 2.2% of control, n = 5; Fig. 5B), verifying that RyRs mediate the long-term potentiation.

### Activation of RyRs increases [Ca^2+^]_i _in DRG neurons and frequency of mEPSCs in superficial dorsal horn neurons

**Figure 6:**
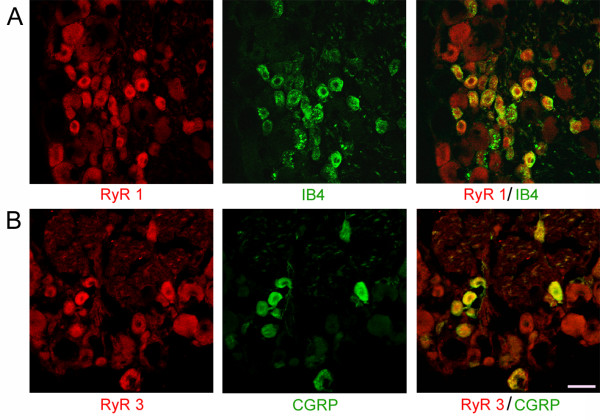
**Expression of ryanodine receptors 1 or 3 (RyR1, RyR3) and their co- localization with IB4 or CGRP in DRG neurons in normal rats**. ***A***, Co-localization of RyR1 (red) with IB4 (green) in DRG neurons. ***B***, Co-localization of RyR3 (red) and CGRP (green) in DRG neurons, scale: 50 μm.

RyRs in the intracellular Ca^2+ ^store have three isoforms: RyR1, RyR2 and RyR3 in the nervous system. As shown in Fig.6, RyR1 and RyR3, but not RyR2, were expressed in DRG neurons. Moreover, RyR1 and RyR3 were co-localized with calcitonin gene-related peptide, (CGRP, marker for peptidergic afferent terminals) and isolectin B4, (IB4, marker for non-peptidergic afferent terminals), respectively.

**Figure 7:**
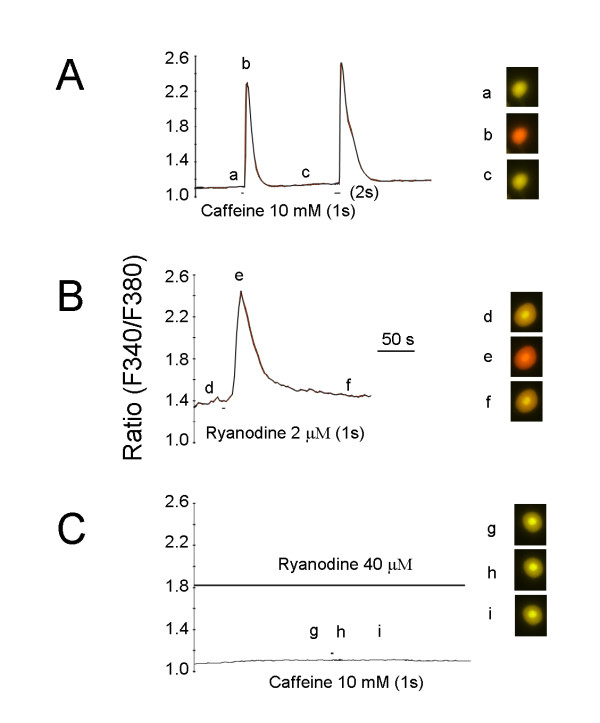
**Increase in [Ca^2+^]_i _by caffeine in isolated DRG neurons**. ***A***, Brief puff of 10 mM caffeine for 1 or 2 s increased [Ca^2+^]_i _transients in DRG neurons (n = 9). ***B***, Puff of 2 μM ryanodine increased [Ca^2+^]_i _transients (n = 9). ***C***, Adding a high concentration of ryanodine (40 μM) to the extracellular solution blocked caffeine-induced increase in [Ca^2+^]_i _transients (n = 6). The cell color imaging (a-i) in the right side corresponded to the time points indicated (a-i) in the left side.

Given RyRs expressed by DRG, the direct action of RyRs agonists was examined by means of calcium imaging in the acute isolated small DRG neurons (15-30 μm). A puff of 10 mM caffeine for 1 or 2 sec increased [Ca^2+^]_i _in 9 DRG neurons tested (Fig. 7A). As mentioned above, low and high concentration of ryanodine functions respectively as agonist and antagonist of RyRs. Perfusion of ryanodine at high concentration (40 μM) 1 h before recording blocked caffeine-increased [Ca^2+^]_i _(Fig. 7C) and a puff of ryanodine at low concentration (2 μM) reversibly elevated [Ca^2+^]_i _(Fig. 7B), unequivocally supporting that DRG neurons express functional RyRs.

**Figure 8:**
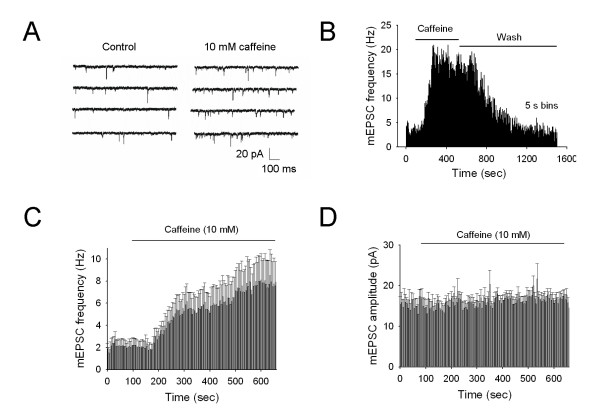
**Increase in mEPSCs frequency by caffeine in superficial dorsal horn neurons**. ***A***, Sample traces of mESPCs recorded from a lamina II neuron in the presence of 0.5 μM TTX before (left) and after bath application of 10 mM caffeine for about 5 min (right). ***B***. A histogram shows the time course of changes in mEPSCs frequency before, after and following washout of 10 mM caffeine in the presence of 0.5 μM TTX in an individual neuron. ***C ***and ***D***, The averaged frequency and amplitude for all cells treated with 10 mM caffeine (n = 16; 5 s bins) in the presence of 0.5 μM TTX. Membrane potential was clamped at -70 mV. Error bars represent s.e.m. n: the number of cells.

Conventionally, the variation of mEPSC frequency reflects the probability of presynaptic transmitter release. In whole-cell patch clamp recording, the effect of caffeine on mEPSC was tested in the superficial laminae of the spinal cord slice with an attached dorsal root. Only those superficial dorsal horn neurons with monosynaptic C-fiber input were selected for recording mEPSCs in the presence of 0.5 μM TTX. Out of total 16 neurons tested, bath application of 10 mM caffeine significantly increased the frequency of mEPSCs in 14 neurons (control: 1.97 ± 0.52 Hz *vs *caffeine: 4.50 ± 1.14 Hz) (n = 16, *P *= 0.0044, Fig. 8A-C), suggesting enhancement of transmitter release probability. But, there was no significant change in the amplitudes of mEPSCs (control: 14.81 ± 1.48 pA *vs *caffeine: 15.49 ± 1.30 pA) (n = 16, *P *= 0.3185, Fig. 8D).

## Discussion

The main findings in the present study are as following: 1, Pharmacological blockade of RyRs prevented the induction of LTP of fEPSPs in the spinal dorsal horn and RyRs agonist alone induced a long-term potentiation; 2, RyRs were expressed in DRG neurons and activation of RyRs elevated [Ca^2+^]_i _in small DRG neurons; 3, Activation of RyRs resulted in a large increase in the frequency of mEPSCs, suggesting a robust increase of presynaptic transmitter release.

These results provided further evidence for the involvement of RyRs in induction of LTP in the spinal pain pathway. Sandkuhler group found that RyR antagonist dantrolene fully blocked low frequency stimulation (LFS)-induced LTP and opioid withdrawal induced-LTP *in vivo *[20,21]. Similarly, the present study showed that RyR antagonist blocked high frequency stimulation (HFS)-induced LTP in the *in vitro *test and titanic sciatic stimulation-induced LTP in the *in vivo *test. It is possible that three different kinds of spinal long-term synaptic plasticity by HFS, LFS and opioid withdrawal may share a similar mechanism of presynaptic activation of RyRs. However, inconsistent with the previous study [22], we detected no significant contribution of IP_3_R for HFS-induced LTP in the spinal dorsal horn *in vitro *(Fig. 4). In support to this, a similar result was obtained in LTP of field potential recordings *in vivo *(data not shown). In their study, blockade of IP_3_R converted LFS-induced LTP into LTD in lamina I spino-PAG neurons, which is similar to our positive control in the hippocampal slices (Fig. 4B). In addition to the different experimental conditions, the contradictory results might be attributed to an involvement of IP_3_R only in LFS- induced LTP in lamina I spino-PAG neurons but not traditional HFS-induced LTP.

The cellular mechanisms underlying RyR-mediated spinal LTP are poorly understood. Our and other evidences suggest that two signal molecules, NO and glutamate, may play a crucial role in RyR function for spinal LTP. Firstly, Previous studies have verified that tetanic stimulation produces a sustained increase in the amounts of glutamate and aspartate from primary C-fiber terminals in the spinal cord [7,23,24] and that blockade of NMDA receptors or the glutamate transporter GLT-1 inhibited the spinal induction of LTP, suggesting a crucial role of glutamate [5]. As shown in Fig. 8A-C, activation of RyRs largely increases in the frequency of mEPSCs in the superficial DH neurons, further supporting enhancement of presynaptic release of transmitters such as glutamate.

Synaptic inputs to the recording dorsal horn neurons mainly derive from two sources: 1) Interneurons of the spinal dorsal horn; 2) Primary afferent terminals. Although we do not further locate the expression of RyRs, and the action of spinal interneurons can not exclude, but combined with the calcium imaging results that activation of RyRs elevated [Ca^2+^]_i _in DRG neurons and RyRs expression by DRG neurons, it is reasonable to speculate that activation of RyRs expressed by presynaptic primary sensory neurons may at least partially involved in the enhancement of glutamate release. Secondly, consistent with hippocampcal LTP [25,26], the diffusible messenger NO also contributes to the induction of spinal LTP [13,14]. It is well documented that NMDA receptor activation induces NO synthesis from arginine [9]. Furthermore, peripheral noxious stimuli or intrathecal glutamate and NMDA induce NO release in the dorsal horn *in vivo *[27]. Intrathecal administration of NMDA causes an increase in cGMP, a production of NO target guanylate cyclase (sGC), in the spinal dorsal horn [28]. Importantly, the RyR is a down-stream target for the NO-PKG-cADPR signaling pathway and PKG-induced production of cADPR synergistically with intracellular Ca^2+ ^activates RyRs [29]. The present result that pre-perfusion of cADPR, an endogenous agonist of the RyR, reversed NOS inhibitor-induced inhibition of spinal LTP of fEPSPs (Fig. 3), supported that activation of RyRs by NO is one of the essential factors for induction of spinal LTP.

Taken together, it is conceivable that glutamate released from presynaptic terminals by conditioning stimulation activates NMDA receptors in the pain-sensitive neurons in the dorsal horn, and then triggers NO synthesis in neurons and glia in the spinal cord. NO retrogradely diffuses from postsynaptic spinal pain-sensitive neurons and glia into presynaptic terminals and activates RyRs releasing Ca^2+^, finally strengthens release of transmitters. Similarly, the putative process also occurs in the hippocampus [30,31].

Although our results emphasized presynaptic action of RyRs in spinal LTP, the postsynaptic function of RyRs is unable to exclude completely due to our limination of methodology. Further more work need to be done.

## Conclusion

Activation of presynptic RyRs were involved in the induction of spinal LTP, probably through enhancement of presynaptic release of neurotransmitters. It is likely that RyR in primary afferent terminals is a novel target for treating pain.

## Materials and methods

*Sprague-Dawley *rats were from Experimental Animal Center, Shanghai Medical College of Fudan University, China. All procedures of the experiments were approved by the Committee of Animal Use for Research and Education of Fudan University, and all efforts were made to minimize the number of animals used and their suffering, *in *accordance with the ethical guidelines for animal research [32].

### Preparation of spinal cord slices

Young Sprague-Dawley rats (postnatal days 14-21) were deeply anesthetized with diethyl ether, and for LTP recording, about 1 ml lidocaine (5 ml:0.1 g) was injected to both sides of lumbar vertebrae (L_4-5_). Laminectomy was performed from mid-thoracic to low lumbar levels and the cord was quickly removed to cold modified artificial cerebrospinal fluid (ACSF): (in mM) NaCl, 80; KCl, 2.5; NaH_2_PO4, 1.25; CaCl_2_, 0.5; MgCl_2_, 3.5; NaHCO_3_, 25; sucrose, 75; ascorbate, 1.3; sodium pyruvate, 3.0; oxygenated with 95% O_2 _and 5% CO_2_; pH 7.4; measured osmolarity 310.5 mOsm. Transverse 500 μm slices, with attached dorsal roots, were obtained. Slices were then incubated for at least 1 h at 35°C in a solution that consisted of: (in mM) NaCl, 125; KCl, 2.5; CaCl_2_, 2; MgCl_2_, 1, NaH_2_PO_4_, 1.25; NaHCO_3_, 26; D-glucose, 25; ascorbate, 1.3; sodium pyruvate, 3.0; oxygenated with 95% O_2 _and 5% CO_2_, pH 7.4; measured osmolarity 324.5 mOsm. The slice was then transferred into a recording chamber and perfused with oxygenated recording solution at a rate of 5 ml min^-1 ^prior to electrophysiological recordings at room temperature.

### Field potential and whole-cell patch clamp recordings from spinal cord slices

Field potential recordings from the superficial spinal dorsal horn were performed with glass microelectrodes (impedance 2-5 MΩ) filled with (in mM) NaCl 135, KCl 5.4, CaCl_2_ 1.8, MgCl_2_ 1, HEPES 5 (pH adjusted to 7.2 with NaOH). A bipolar tungsten electrode was used to stimulate LT. Low-pass filter was set to 1 kHz, amplification 500× (Axopatch 200B, Axon Instruments). To minimize current spread to the dorsal roots and the recording site, the electrode was placed at the most ventrolateral border of LT [33]. For LTP associated experiments, recordings of fEPSPs were made in the presence of bicuculline (10 μM) and strychnine (1 μM) to block tonic inhibitory action of GABA_A _and glycine receptors and data are normalized (to mean values of 10 min before HFS or caffeine exposure) mean peak fEPSPs amplitudes. In some of the experiments (caffeine associated), a suction electrode (A-M systems) was used for electrical stimulation of the attached dorsal root. Neurons were identified by infrared differential interference contrast (IR-DIC) video microscopy with an upright microscope (Leica DMLFSA, Germany) equipped with a 40×, 0.80 NA water-immersion objective and a CCD camera (IR-1000E, USA). Patch pipettes (5-10 MΩ) from borosilicate glass were made on a horizontal micropipette puller (P-97, Sutter Instruments, Novato, CA, USA) and were filled with (in mM) potassium gluconate 120, KCl 20, MgCl_2_ 2, Na_2_ATP 2, NaGTP 0.5, HEPES 20, EGTA 0.5, pH 7.28 with KOH, measured osmolarity 300 mM. To measure EPSCs from neurons in the inner part of lamina II, the dorsal root was stimulated via a suction electrode. Test pulses of 0.1 msec (0.7-1 mA) were given at 30 sec intervals. Data were acquired using an Axopatch 200B patch-clamp amplifier. Responses were low-pass filtered on-line at 2 kHz, digitized at 5 kHz, and analyzed off-line using Clampfit 8.1 software. Membrane potential was held at -70 mV and all recordings were performed at room temperature.

### Field potential recording from hippocampal slices

Young Sprague-Dawley rats (postnatal days 14-21) were deeply anesthetized with diethyl ether. The brain was quickly removed and immersed in ice-cold ACSF bubbled with a gas mixture of 95% O_2 _and 5% CO_2_. 400 μM transverse slices were prepared. The slices were then incubated in a chamber maintained at 35°C for at least 1.5 h before recording, and a single slice was then transferred into a recording chamber and perfused with oxygenated recording solution at 3 ml min^-1 ^prior to electrophysiological recordings at room temperature. The composition of the ACSF was (in mM): 124 NaCl, 4.4 KCl, 2.5 CaCl_2_, 1.3 MgSO_4_, 1 NaH_2_PO_4_, 26 NaHCO_3_, and 10 glucose. Field potential recordings were performed as previously described [15]. Briefly, fEPSPs were recorded with a glass micropipette containing 1 M NaCl in the stratum radiatum of the CA1 region, stimulated *via *a bipolar tungsten electrode placed along the Schaffer collateral fibers. The stimulation intensity was adjusted to produce a half-maximal field potential amplitude at the beginning of each experiment. Test pulses of 0.1 msec were given at 100 sec intervals. To induce LTP, HFS (100 Hz for 1 sec, 3 times at 10-sec intervals) was delivered, similar to the induction protocol for spinal LTP *in vitro*.

### Recording of C-fiber-evoked field potentials in vivo

As we described previously [14], experiments were performed on adult male Sprague-Dawley rats weighing 250-300 g. Pairs of rats were housed in plastic cages and maintained on a 12:12 h light-dark cycle and constant room temperature of 21°C with free access to food and water. Urethane (1.5 g/kg, intraperitoneally) was used to induce and maintain anesthesia. Surgical level of anesthesia was verified by the absence of corneal reflexes and foot withdrawal to pinch. The trachea was cannulated to allow mechanical ventilation with room air, if necessary. One carotid artery was cannulated to monitor mean arterial blood pressure, which ranged from 80 to 100 mmHg. A laminectomy was performed at vertebrae T_13_-L_1 _to expose the lumbar enlargement, and the left sciatic nerve was dissected free for bipolar electrical stimulation with a pair of stainless-steel hook electrodes. The exposed nervous tissues were covered with warm paraffin oil. An intrathecal catheter (PE-10 tube) was inserted through the gap between the L_4 _and L_5 _vertebrae and extended to the subarachniod space of the lumbar enlargement (L_4 _and L_5 _segments). Colorectal temperature was kept constant at 37-38°C by a feedback-controlled heating blanket. The electrocardiogram was monitored continuously.

Following electrical stimulation of the sciatic nerve, the field potentials were recorded in the ipsilateral L_4-5 _segments, 300-800 μm from the surface of the cord with glass microelectrodes (impedance 3-6 MΩ) which were driven by an electronically controlled microstepping motor (Narishige Scientific Instrument Co.). The low-pass filter was adjusted to 100 Hz. An A/D converter card (SUMP-PC, Shanghai Medical College, Fudan University, China) was used to digitize and store data at 10 kHz. A single rectangular pulse (0.5 ms, 15-20 V), sufficient to excite C afferent fibers, was applied to sciatic nerve at 1 min intervals as test stimulus. It was easy to distinguish the C-fiber-evoked field potentials from A-fiber-evoked potentials by the latencies of the A and C responses. After recording stable responses for more than 40 min, conditioning tetanic stimuli (0.5 m, 100 Hz, 30-40 V, 10 trains of 2 sec duration at 10 sec intervals) were delivered to the sciatic nerve for inducing LTP of C-fiber-evoked field potentials. The distance from the stimulation site to the recording site was approximately 10 cm. At the end of experiments, rats were killed by an overdose of urethane.

The area of C-fiber-evoked field potentials was determined off-line by Average Soft provided by the Department of Physiology, Shanghai Medical College, Fudan University. In each experiment, responses to six consecutive test stimuli were averaged. Data are normalized to mean values of the first 30 min areas of C-fiber evoked field potentials.

### Calcium imaging

As we previously described [34], acutely isolated DRG neurons were loaded with 1 μM Fura-2 acetoxymethyl ester (Fura-2/AM; DoJinDo Laboratories, Kumamoto, Japan). The neurons were observed on an inverted microscope (Olympus IX51) with a 40× UV flour oil-immersion objective lens. The fluorescence in individual neurons was recorded by a cooled CCD camera (Hamamatsu, Hamamatsu City, Japan), with a 1 Hz alternating wavelength time scanning with excitation wavelengths of 340 and 380 nm and an emission wavelength of 510 nm (monochromators; Till Polychrome IV, Munich, Germany). Images were captured every 3 sec. Digitized images were acquired and analyzed in a personal computer controlled by SimplePCI (Compix, Lake Oswego, OR). The ratio of the fluorescence at the two excitation wavelengths was used to estimate changes of [Ca^2+^]_i_.

### Immunohistochemistry

Normal adult rats were given an overdose of urethane (2 g/kg, i.p.) and perfused intracardially with 0.9% sterile saline followed by 4% paraformaldehyde in 0.1 M phosphate buffer (PB, pH 7.4). The L_4-6 _DRG were then removed, post-fixed in the same fixative for 2 h at 4°C, and immersed in 20% sucrose in PB for 24 h at 4°C for cryoprotection. Transverse DRG sections (14 μm) were cut in a cryostat and processed for immunofluorescence. All sections were blocked with 10% donkey serum in 0.01 M PBS, pH 7.4 with 0.3% Triton-X-100 for 2 h at room temperature (RT) and incubated 24 h at 4°C with rabbit anti-RyR 1 and 3 antibody (1: 1000, Chemicon, Temecula, CA) or a mixture of rabbit anti-RyR 1 and 3 antibody (1: 1000, Chemicon, Temecula, CA) and goat anti-CGRP antibody (1:500, Santa Cruze) in PBS with 1% normal donkey serum and 0.3% Triton-X-100. Following six 10 min rinses in 0.01 M PBS, the sections were incubated in a mixture of FITC-conjugated Isolectin B4 (IB4) (1:1000, Sigma) and rhodamine red-X-conjugated donkey anti-rabbit IgG (1:100, Jackson ImmunoResearch, West Grove, PA) for 2 h at 37°C, or FITC-conjugated donkey anti-goat IgG (1:100, Jackson ImmunoResearch, West Grove, PA) and rhodamine red-X-conjugated donkey anti-rabbit IgG (1:100, Jackson ImmunoResearch, West Grove, PA) for 120 min at RT, and then washed in PBS. The specificity of immunostaining was verified by omitting the primary antibodies, and immunostaining signal disappeared after omitting primary antibodies. All sections were coverslipped with a mixture of 50% glycerin in 0.01 M PBS, and then observed with a Leica fluorescence microscope, and images were captured with a CCD spot camera.

### Drugs

*In vivo *experiments: Ryanodine was initially dissolved in dimethylsulfoxide (DMSO) and then diluted in physiological saline to their final concentration immediately before administration. The final DMSO concentration in the diluted working solution was 0.25%. To evaluate the effects of drugs on spinal LTP induction and maintenance, drugs or vehicles were intrathecally injected over a period of 2 min at a volume of 10 μl followed by 5 μl 0.9% sterile saline solution for flushing at 30 min before or 60 min after tetanic stimuli. *In vitro *experiments: Most of the drugs were purchased from sigma, including bicuculline methiodide (10 μM), strychnine (1 μM), AP5 (50 μM), DNQX (10 μM), dantrolene (10 μM), 2-APB (75 μM), cADPR (5 μM), L-NAME (50 μM). The other drugs were TTX (0.5 μM), ryanodine (2 μM, 20 μM, 20 μM, Calbiochem) and caffeine (10 mM, Lancaster). All drugs were prepared as stock solutions and diluted to the required concentration (1:1000) with ACSF.

### Statistics

Data were expressed as mean ± s.e.m. Effects of drugs on LTP were compared with two-way repeated measures (RM) ANOVA (treatment and time) followed by the Holm-Sidak tests. Pared *t*-test or *t*-test was used for other electrophysiology experiments. *P *< 0.05 was considered statistically significant.

## Competing interests

The authors declare that they have no competing interests.

## Authors' contributions

LZC performed electrophysiological experiments in the spinal cord slice and calcium imaging in DRG neurons and drafted the manuscript. NL performed the *in vivo *electrophysiological and immunochemical experiments. YQZ was involved in experimental design and guiding. ZQZ designed the experiments and revised the manuscript. All authors have read and approved the final manuscript.
